# Nutrition advice aimed at children also improves parents’ diets

**Published:** 2018

Nutrition advice aimed at children also improves parents’ diets, according to research published recently in the *European Journal of Preventive Cardiology*.

‘Diets high in unsaturated fat and low in saturated fat have been associated with a reduced risk of cardiovascular events and death in adults,’ said lead author Dr Johanna Jaakkola, a postdoctoral researcher at the University of Turku, Finland. ‘Very little is known about the long-term effects of nutrition advice for children on the diets and health of parents.’

The longitudinal randomised Special Turku Coronary Risk Factor Intervention Project (STRIP) decreased the saturated fat intake and improved the cardiovascular health of children by recommending foods rich in unsaturated instead of saturated fat. The current study examined whether the long-term dietary intervention focused on children was also associated with parental dietary intake and cardiometabolic risk factors over two decades of follow up.

The primary results of the STRIP study have been previously reported. Briefly, the study included 1 107 infants and their parents who were recruited from well-baby clinics in Turku, Finland, between 1989 and 1992. Families were randomly assigned to the dietary intervention (562) or control (545) groups.

The intervention group received dietary counselling at least once a year by a nutritionist from the child’s age of eight months to the age of 20 years. Counselling was first given only to the parents, and from the age of seven years, the children were also met alone. The main focus of the dietary intervention was to reduce the child’s intake of saturated fat and concomitantly increase the child’s unsaturated fat intake.

As previously reported, the repeated dietary counselling led to decreased saturated fat intake in the intervention children, and lower serum low-density lipoprotein (LDL) cholesterol concentration from infancy until 19 years of age.

For the current study, parental dietary intake was assessed by a one-day food record biennially from the child’s age of nine to 19 years. Weight and height, and blood pressure, serum lipid, glucose and insulin levels of the parents were measured repeatedly from the child’s age of seven months until 20 years.

The investigators found that the child-oriented dietary counselling increased the intake of polyunsaturated and monounsaturated fats and decreased the saturated fat intake of intervention mothers and fathers compared to control parents between the child’s ages of nine and 19 years.

In addition, the child-oriented dietary counselling tended to decrease serum total and LDL concentrations in intervention mothers compared to control mothers. There was a similar trend in fathers but it was not statistically significant.

Dr Jaakkola said: ‘The child-oriented dietary intervention contributed advantageously to the parental diet in the long term and tended to reflect lipid concentrations, particularly in mothers. Presumably all family members eat the same foods and therefore child-oriented dietary counselling also affects parents’ diets.’

‘“Dietary intake may have been more strongly associated with maternal than paternal serum lipid levels because mothers might have more actively participated in the study and complied better with the diet,’ she continued. ‘There is also the possibility that the improvement in the fathers’ diets was not strong enough to cause a statistically significant difference in serum lipid levels.’

Dr Jaakkola concluded: ‘Our study emphasises that long-term dietary counselling directed at children may be an efficient way to also improve the diets of parents. These findings could be used to plan public health counselling programmes.’
